# Profiling metabolites of *Ficus natalensis* hochst. fruit by UPLC-MS/MS and evaluation of anti-inflammatory activity

**DOI:** 10.1038/s41598-026-51688-4

**Published:** 2026-05-13

**Authors:** Enas M. Shawky, Rim Hamdy, Mostafa H. Baky

**Affiliations:** 1 Department of Pharmacognosy, Faculty of Pharmacy, Egyptian Russian University, Badr City, Cairo, 11829 Egypt; 2 Botany and Microbiology Department, Faculty of Science, Cairo University, Giza, 12613 Egypt; 3 Department of Biological Sciences, Faculty of Science, Galala University, New Galala City, Suez, 43511 Egypt

**Keywords:** Moraceae, *Ficus natalensis*, UHPLC-MS/MS, Anti-inflammatory activity, Biochemistry, Chemistry, Drug discovery, Plant sciences

## Abstract

*Ficus natalensis* (Natal fig), an evergreen tree of the family Moraceae, is widely distributed across tropical and temperate regions and cultivated in Egypt. Members of the Moraceae family are traditionally employed as expectorants, hypoglycemic agents, and mild laxatives, with reported neuroprotective, analgesic, anti-inflammatory, and antihypertensive effects. The present study aimed to profile the secondary metabolites of *F. natalensis* fruit using ultra-performance liquid chromatography coupled with tandem mass spectrometry (UPLC-MS/MS) and test its anti-inflammatory potential. A total of 160 metabolites were annotated in both positive and negative ionization modes, belonging to diverse phytochemical classes such as phenolics (41), flavonoids (21), acids (26), glycosides (11), terpenoids, coumarins (4), iridoids (3), fatty acids/ester (22), sterols (8), sugar derivatives (6), and terpenoids (18). The anti-inflammatory activity of *F. natalensis* fruit methanolic extract was investigated using nitric acid (NO) inhibition assay, revealing an IC_50_ of 28.54 ± 1.66 µg/mL, compared to resveratrol as a standard anti-inflammatory with an IC_50_ value of 10.21 ± 0.68 µg/mL. Major bioactive constituents, including ellagic acid, gallic acid, betulinic acid, and quercetin derivatives, were identified and may underline the observed anti-inflammatory effect. The current findings highlight *F. natalensis* fruit as a rich source of bioactive metabolites with potential anti-inflammatory effect.

**Supplementary Information:**

The online version contains supplementary material available at 10.1038/s41598-026-51688-4.

## Introduction

Plants are considered the cornerstone of human nutrition and medicine, serving as vital sources of bioactive compounds with high efficacy and safety. Unlike synthetic drugs, which often produce undesirable side effects, plant-derived metabolites such as alkaloids, glycosides, terpenoids, and flavonoids offer diverse therapeutic benefits and play crucial roles in maintaining human health^1^.

The Moraceae family, commonly known as the fig or mulberry family, encompasses flowering plants of considerable biological and therapeutic significance. Members of this family exhibit a wide spectrum of pharmacological activities, including anti-inflammatory, anticancer, antimicrobial, and gastroprotective effects^2^. Beyond their medicinal roles, Moraceae species are widely utilized in the food, cosmetic, pharmaceutical, and agricultural industries. The family comprises approximately 38 genera and 1,100 species distributed across tropical and subtropical regions, with *Ficus* representing the largest genus, containing more than 850 species of trees, shrubs, and vines^3^. The genus *Ficus* holds exceptional nutritional and medicinal importance, providing edible fruits and phytochemically rich plant parts^4^. They originated in tropical habitat types, with about 100 species originating in African countries and nearby islands. Moreover, these species have been used in traditional medical practices as hypoglycaemic, laxative, anti-rheumatic, anthelmintic, and antihypertensive agents, and also for inflammation, dyspepsia, stomach-aches, and dysentery^5,6^.

The Natal fig (*Ficus natalensis* Hochst.) is an evergreen species cultivated in Egypt primarily for ornamental and environmental purposes. Beyond its landscape value, the species is increasingly recognized as a rich reservoir of phytochemicals, including flavonoids, tannins, and triterpenoid saponins, which contribute to its therapeutic potential^3^. Previous biologically guided studies on *F. natalensis* leaves have demonstrated potent antioxidant, antimicrobial, cytotoxic, and anti-inflammatory activities^12,13^.

In recent years, metabolomics tools are widely applied for phytochemical profiling of natural products^14^. Among these tools, UPLC-MS/MS technique is well suited for profiling of non-volatile metabolites among plant samples including fruits and vegetables^15^. LC-MS/MS was applied for profiling of several *Ficus* species including *F. deltoidei*, *F. drupacea* and *F. sycomorus*^16^, *F. benghalensis* aerial root^17^, and *F. lyrata* leaves and fruits^18^.

The main goal of the current study was to profile the secondary metabolites of *F. natalensis* fruits using UPLC-MS/MS and to correlate the resulting chemical profile with its anti-inflammatory activity.

## Results and discussion

**Fig. 1:**
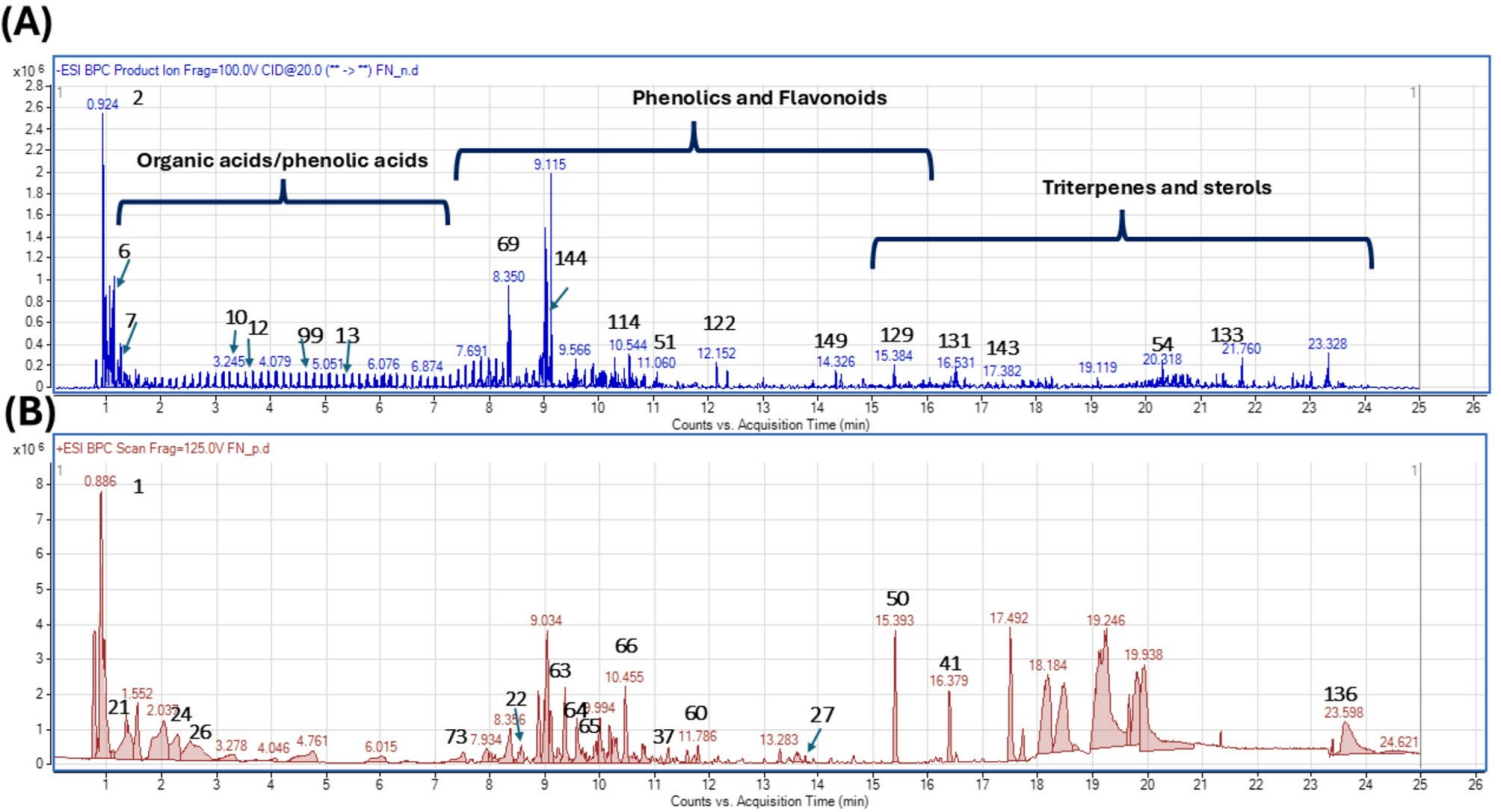
UPLC-MS base peak chromatograms of *Ficus natalensis* fruits **A**) BPC in negative mode **B**) BPC in positive mode. Peaks are numbered according to Table 1, corresponding to putatively annotated metabolites.

**Fig. 2:**
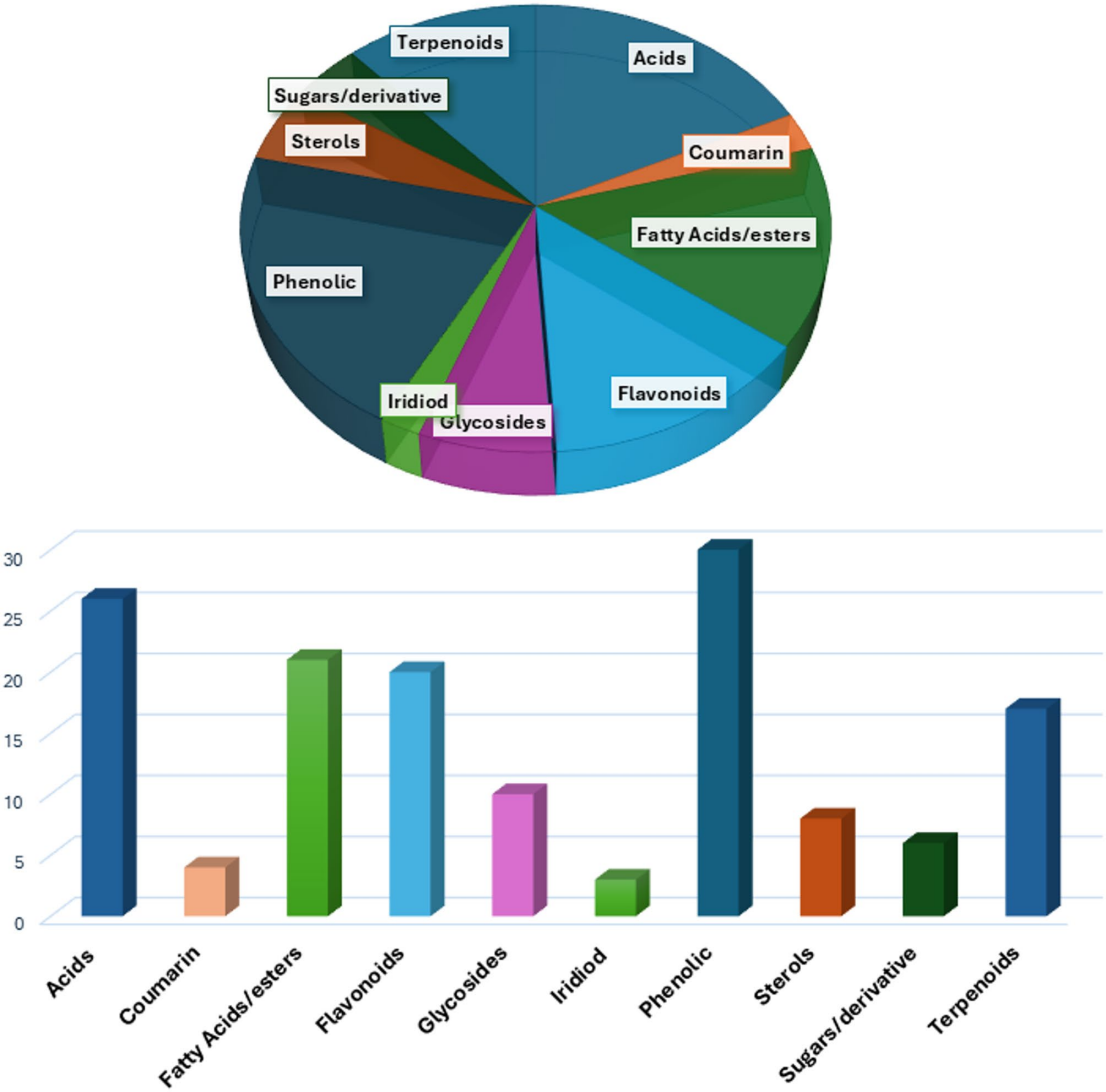
Representative bar-chart and pie chart of identified metabolites classes in *F. natalensis* fruits using UPLC-MS negative and positive modes based on number of compounds identified in each class.

**Fig. 3:**
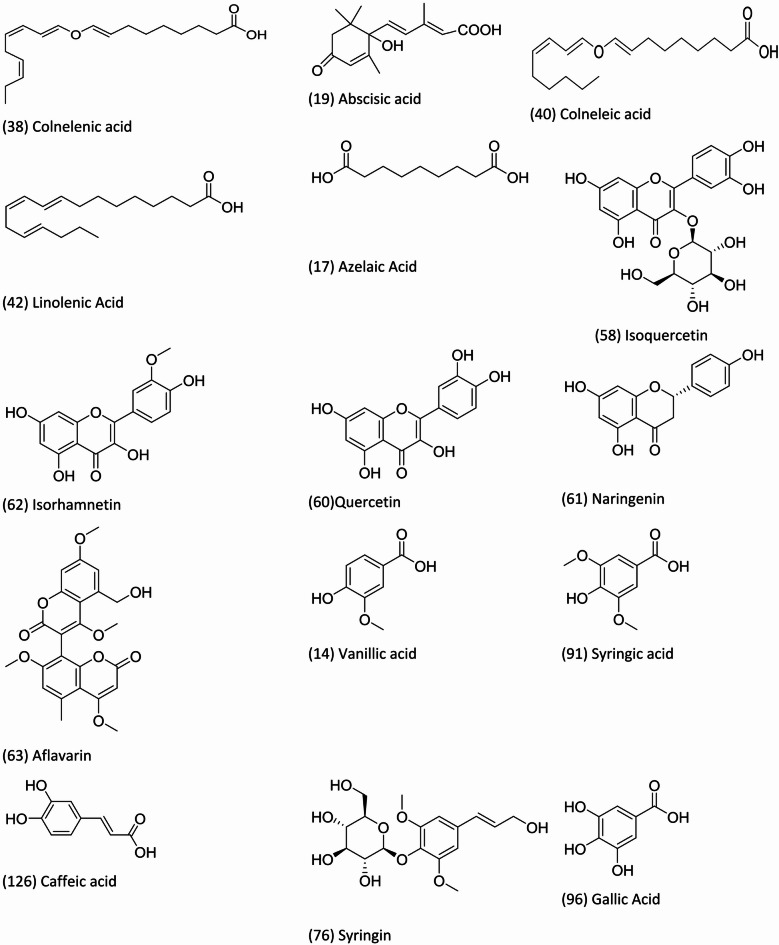

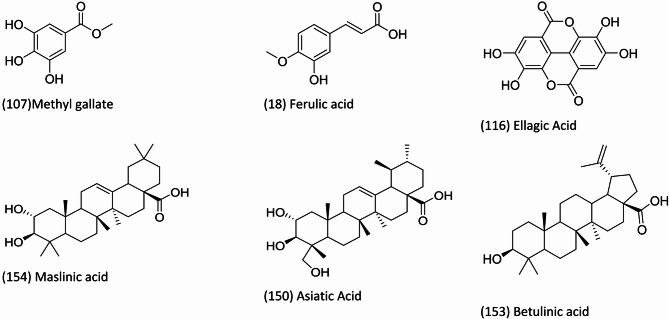
The chemical structure of selected phytochemicals identified in *F. natalensis* fruit extract using UPLC-MS/MS analysis, the name and number of the compounds as listed in Table 1.

Metabolites profiling of *F. natalensis* fruit extract using UPLC-MS/MS in both positive and negative ionization modes (Fig. 1) led to the identification of 160 metabolites belonging to different phytochemical classes, including phenolics (41), flavonoids (21), acids (26), glycosides (11), terpenoids, coumarins (4), iridoids (3), fatty acids/ester (22), sterols (8), sugar derivatives (6), and terpenoids (18) (Fig. 2). Compound identification was achieved by comparing their molecular ions ([M-H]^−^ or [M + H]^+^), retention times (Rt), characteristic fragment ions (Figure S1-S6), and reference spectra from the literature. The chemical structures of selected phytochemicals identified in *F. natalensis* fruit extract are illustrated in Fig. 3.

**Table 1:** Chemical Metabolites tentatively identified in *F. natalensis* fruits by UPLC-MS/MS at negative and positive modes.

No	*R*_T_	Mass (MW)	m/z [M-H]⁻ m/z [M + H]⁺	Formala	Cpd name	Class	Fragmentation	Error	References
Acids									
1.	0.889	226.068	225.0607	C_7_ H_14_ O_8_^−^	Glucoheptonic acid	Acid	215,165,135, 89	0.86	
2.	0.902	166.0471	165.0398	C_5_ H_10_ O_6_^−^	Arabonic acid	Acid	133	0.65	
3.	1.075	354.0936	353.0863	C_16_ H_18_ O_9_^−^	3-O-Caffeoyl quinic acid	Acid	191	1.5	^30^
4.	1.214	476.1355	475.128	C_16_ H_28_ O_16_^−^	Malic acid dihexoside	Acid	133	2.28	^67^
5.	1.258	192.0626	191.0553	C_7_ H_12_ O_6_^−^	Quinic acid	Acid	133, 93	0.79	^68^
6.	1.312	134.021	133.0137	C_4_ H_6_ O_5_^−^	Malic acid	Acid	115, 71	0.55	^69^
7.	1.477	192.0263	191.019	C_6_ H_8_ O_7_^−^	Citric acid	Acid	111, 87, 76, 57	0.72	^69^
8.	1.5	112.0155	111.0083	C_5_ H_4_ O_3_^−^	Furoic acid	Acid	-	0.51	^70^
9.	1.809	148.0365	147.0292	C_5_ H_8_ O_5_^−^	Citramalic acid	Acid	113	0.67	^37^
10.	3.284	132.0416	131.0344	C_5_ H_8_ O_4_^−^	Dimethyl malonate	Acid	130, 91, 70	0.62	^37^
11.	3.482	330.094	329.0868	C_14_ H_18_ O_9_^−^	Vanillic acid-4-O-β-D-glucopyranoside	Acid	167, 122	1.1	^71^
12.	3.65	206.0419	205.0346	C_7_ H_10_ O_7_^−^	Homocitric acid	Acid	153, 111	0.78	^37^
13.	5.315	316.0779	315.0707	C_13_ H_16_ O_9_^−^	Dihydroxybenzoic acid hexoside I	Acid	153, 109	1.51	^72^
14.	5.733	168.0415	167.0342	C_8_ H_8_ O_4_^−^	Vanillic acid	Acid	122, 108	0.75	^71^
15.	7.591	138.0308	137.0235	C_7_ H_6_ O_3_^−^	P-Hydroxybenzoic acid	Acid	137, 93	0.89	^73^
16.	7.92	176.0676	175.0604	C_7_ H_12_ O_5_^−^	2-Isopropylmalic acid	Acid	137, 115, 113	0.84	^71^
17.	10.982	188.104	187.0968	C_9_ H_16_ O_4_^−^	Azelaic Acid	Acid	169, 125, 97	0.83	^74^
18.	11.295	194.0572	193.05	C_10_ H_10_ O_4_^−^	Ferulic acid	Acid	149	0.68	^25^
19.	11.781	264.1351	263.1278	C_15_ H_20_ O_4_^−^	Abscisic acid	Acid	145, 117	1.09	^37^
20.	11.817	202.1197	201.1124	C_10_ H_18_ O_4_^−^	Sebacic Acid	Acid	183, 139	0.85	^75^
21.	12.6	216.1352	215.1279	C_11_ H_20_ O_4_^−^	Undecanedioic Acid	Acid	197, 153	1	^76^
22.	8.638	374.1187	375.1263	C_16_ H_22_ O_10_^+^	Geniposidic acid	Acid	282, 207, 159	2.57	^77^
23.	1.103	364.0983	365.1056	C_14_ H_20_ O_11_^+^	2,3,4,5-Tetra-O-acetylhexonic acid	Acid	176, 157, 118, 101	2.25	^78^
24.	2.164	156.04	157.047	C_7_ H_8_ O_4_^+^	Heptadienedioic acid	Acid	132, 108	2.49	^79^
25.	2.65	140.009	141.0158	C_6_ H_4_ O_4_^+^	Coumalic acid	Acid	141, 101	2.45	
26.	2.69	118.026	119.0337	C_4_ H_6_ O_4_^+^	Succinic Acid	Acid	119, 101	0.17	
Coumarins									
27.	13.919	262.048	263.0561	C_13_ H_10_ O_6_^+^	Maclurin	Coumarin	223, 100	0.09	^37^
28.	1.257	380.072	381.0799	C_17_ H_16_ O_10_^+^	5-O-β-Dglucopyranosyl-6- hydroxyangelicin	Coumarin	381, 219, 201	2.17	^80^
29.	8.78	338.0987	337.0915	C_16_ H_18_ O_8_^−^	P-Coumaroylquinic acid	Coumarin	163, 191	1.46	^81^
30.	14.1	386.1345	385.1273	C_21_ H_22_ O_7_^−^	Pteryxin	Coumarin	238, 112	2.03	
Fatty acids									
31.	13.322	288.2284	287.2211	C_16_ H_32_ O_4_^−^	Dihydroxyhexadecanoic acid	Fatty acid	287, 269	1.66	^82^
32.	13.704	328.2232	327.216	C_18_ H_32_ O_5_^−^	9,12,13-Trihydroxy-10,15- octadecadienoic acid	Fatty acid	309, 291, 239, 229, 221, 211, 191, 183, 171, 137	1.81	^82^
33.	13.969	308.1972	307.1899	C_18_ H_28_ O_4_^−^	16-Hydroxy-9-oxo-10E,12E,14E-octadecatrienoic acid	Fatty acid	289, 235, 211, 209, 185, 121	1.58	^83^
34.	14.005	330.239	329.2317	C_18_ H_34_ O_5_^−^	9,12,13-Trihydroxy-10-octadecenoic acid	Fatty acid	229, 211, 171	1.61	^82^
35.	15.17	310.2128	309.2055	C_18_ H_30_ O_4_^−^	Unknown Fatty acid	Fatty acid	238, 112	1.61	
36.	11.08	414.259	415.2663	C_22_ H_38_ O_7_^+^	Ascorbyl palmitate	Fatty acid	371, 115	2.73	^84^
37.	11.336	228.134	229.1411	C_12_ H_20_ O_4_^+^	Dodecenedioic acid	Fatty acid	183, 165	2.4	^82^
38.	12.624	292.204	293.2107	C_18_ H_28_ O_3_^+^	Colnelenic acid	Fatty acid	195, 100	0.39	^37^
39.	12.973	212.1402	213.146	C_12_ H_20_ O_3_^+^	Dihydrojasmonic acid	Fatty acid	195, 100	2.48	^37^
40.	13.047	294.2188	295.2262	C_18_ H_30_ O_3_^+^	Colneleic acid	Fatty acid	195, 100	0.76	^37^
41.	16.173	276.209	277.2161	C_18_ H_28_ O_2_^+^	Stearidonic Acid	Fatty acid	235, 221, 207	-0.05	^14^
42.	16.798	278.2246	279.2319	C_18_ H_30_ O_2_^+^	Linolenic Acid	Fatty acid	144, 137	-0.05	^85^
43.	16.766	296.2339	295.2265	C_18_ H_32_ O_3_^−^	13-Hydroxyoctadeca-9,15-dienoic acid	Fatty acid	295, 277, 183	1.25	^82^
44.	17.532	298.2495	297.2423	C_18_ H_34_ O_3_^−^	Ricinoleic acid	Fatty acid	194	1.3	^37^
45.	18.086	300.2652	299.2579	C_18_ H_36_ O_3_^−^	2-Hydroxyethyl palmitate	Fatty acid	238, 155, 112	1.24	
46.	18.967	272.2341	271.2268	C_16_ H_32_ O_3_^−^	12-hydroxy-13-methyltetradecanoate	Fatty acid	248, 155, 112	1.01	^86^
47.	20.331	328.2965	327.2892	C_20_ H_40_ O_3_^−^	2-Hydroxybutyl palmitate	Fatty acid	144, 100	1.26	
48.	22.146	400.3534	399.346	C_24_ H_48_ O_4_^−^	Monoheneicosanoin	Fatty acid	355, 316, 125, 100	1.83	
49.	22.205	356.3278	355.3205	C_22_ H_44_ O_3_^−^	2-Hydroxydocosanoic acid	Fatty acid	316, 125, 100	1.24	
50.	15.392	352.2617	353.2692	C_21_ H_36_ O_4_^+^	Glyceryl linolenate	Fatty acid	304, 221,100	-0.35	^37^
51.	11.112	392.2756	391.2683	C_20_ H_40_ O_7_^−^	D-Glucitol monomyristate	Fatty acid ester	187, 137	1.81	
52.	20.093	314.2809	313.2736	C_19_ H_38_ O_3_^−^	Methyl 2-hydroxystearate	Fatty acid ester	239, 125, 100	1.24	
Flavonoids									
53.	11.729	550.1662	549.1589	C_26_ H_30_ O_13_^−^	Liquiritin apioside	Flavanoids	255, 135	2.44	^87^
54.	20.31	356.1641	355.1572	C_21_ H_24_ O_5_^−^	Unknown flavanoid	Flavanoids	327, 255, 144	-1.68	
55.	15.656	370.1398	369.1325	C_21_ H_22_ O_6_^−^	Sophoraisoflavanone A	Flavanoids	369,208, 161, 135	1.83	^88^
56.	9.803	596.135	595.1278	C_26_ H_28_ O_16_^−^	Quercetin-3-O-pentosylhexoside	Flavanoids	300, 271, 179	2.78	^89^
57.	10.115	610.1505	609.1433	C_27_ H_30_ O_16_^−^	Kaempferol dihexoside	Flavanoids	447, 285, 197, 153	2.84	^28^
58.	10.278	464.0937	463.0864	C_21_ H_20_ O_12_^−^	Isoquercetin	Flavanoids	301, 271, 255	1.74	^90^
59.	10.752	484.025	483.0178	C_22_ H_12_ O_13_^−^	Methyl (S)-flavogallonate	Flavanoids	450, 407, 299	2.79	^91^
60.	11.885	302.0413	301.034	C_15_ H_10_ O_7_^−^	Quercetin	Flavanoids	153, 181	1.33	^92^
61.	12.541	272.0673	271.06	C_15_ H_12_ O_5_^−^	Naringenin	Flavanoids	151, 177	1.21	^93^
62.	12.853	316.057	315.0497	C_16_ H_12_ O_7_^−^	Isorhamnetin	Flavanoids	300	1.3	^94^
63.	9.3	454.1268	455.1341	C_24_ H_22_ O_9_^+^	Aflavarin	Flavanoids	281, 195	-0.43	^37^
64.	9.748	404.1649	405.1736	C_25_ H_24_ O_5_^+^	Osajin	Flavanoids	319, 279, 251, 223, 137	-2.56	^95^
65.	9.834	492.1243	493.1315	C_23_ H_24_ O_12_^+^	Tricin O-hexoside	Flavanoids	331	2.46	^96^
66.	10.556	434.085	435.0923	C_20_ H_18_ O_11_^+^	Avicularin	Flavanoids	301	-0.08	^97^
67.	12.207	248.1022	249.1095	C_14_ H_16_ O_4_^+^	Prenyl caffeate	Flavanoids	226, 100	2.65	^37^
68.	8.233	666.1763	665.1687	C_30_ H_34_ O_17_^−^	Chrysoeriol-O-acetylglucoside-O-glucoside	Flavanoids	443, 353, 315, 285	3.3	^98^
69.	8.372	708.1872	707.1801	C_32_ H_36_ O_18_^−^	Kalambroside A	Flavanoids	443, 353, 285	2.99	^99^
70.	8.58	452.223	451.2159	C_27_ H_32_ O_6_^−^	Kushenol D	Flavanoids	443, 353, 234, 183	-3.13	^88^
71.	8.97	696.1881	695.1812	C_31_ H_36_ O_18_^−^	Apigenin C-pentoside-C- hydroxyferuloyl-pentoside	Flavanoids	353, 341	2.03	^100^
72.	3.789	414.0925	415.1003	C_21_ H_18_ O_9_^+^	Flavan-3-ol-(4 − 2)- phloroglucinol	Flavanoids	289, 139, 121	2.59	^101^
73.	7.39	436.1557	437.1628	C_25_ H_24_ O_7_^+^	Artonin J	Flavanoids	429, 370, 311, 226	-3.53	^102^
Glycosides									
74.	3.828	324.082	325.0894	C_15_ H_16_ O_8_^+^	Umbelliferone glucoside	Glycosides	280, 163, 119, 107	2.48	^47^
75.	8.657	414.1499	415.1573	C_19_ H_26_ O_10_^+^	Ptelatoside A	Glycosides	371, 238	2.66	
76.	10.474	372.1419	373.1494	C_17_ H_24_ O_9_^+^	Syringin	Glycosides	371,209	0.08	^48^
77.	8.094	444.1977	443.1905	C_21_ H_32_ O_10_^−^	Cynaroside A	Glycosides	443, 353	1.8	^103^
78.	9.289	416.1667	415.1591	C_19_ H_28_ O_10_^−^	Phenethyl primeveroside	Glycosides	337, 223, 175, 151	1.57	^28^
79.	10.185	442.1826	441.1752	C_21_ H_30_ O_10_^−^	(1′S, 6′R)-8’-Hydroxyabscisic acid β-D-glucoside	Glycosides	415, 371	1.33	^104^
80.	10.462	472.1925	471.1851	C_22_H_32_ O_11_^−^	Eugenol rutinoside	Glycosides	163, 148	1.96	^49^
81.	10.748	394.1817	393.1746	C_17_ H_30_ O_10_^−^	3-Hexenyl b-primeveroside	Glycosides	250, 99, 57	2.24	^105^
82.	10.783	416.2028	415.1956	C_20_ H_32_ O_9_^+^	Ethyl 7-epi-12-hydroxyjasmonate glucoside	Glycosides	389/405	1.81	^106^
83.	10.869	420.1395	419.1322	C_21_ H_24_ O_9_^−^	Rhapontigenin O-glucoside	Glycosides	401, 299, 281, 257, 241, 225	2.58	^107^
84.	11.273	402.1498	401.1433	C_18_ H_26_ O_10_^−^	Benzyl beta-primeveroside	Glycosides	289, 269, 193	2.76	^108^
Iridoids									
85.	1.692	434.1403	435.1477	C_18_ H_26_ O_12_^+^	8-O-Acetylshanzhiside	Iridiod	365, 229, 182	2.12	^43^
86.	1.051	696.2087	695.2019	C_28_ H_40_ O_20_^−^	Monotropein dihexoside	Iridoid glycosides	533, 383, 353, 191, 133	2.61	^45^
87.	2.816	452.1504	451.1433	C_18_ H_28_ O_13_^−^	Shanzhiside methyl ester	Iridoid glycosides	243, 101	2.6	^44^
Phenolics									
88.	1.186	214.0453	215.0526	C_9_ H_10_ O_6_^+^	2-Hydroxyethyl gallate	Phenolic	176, 141	2.41	^37^
89.	1.993	164.0474	165.0546	C_9_ H_8_ O_3_^+^	3’-Hydroxycinnamic acid	Phenolic	132, 119	-0.05	
90.	3.749	332.1076	333.1154	C_14_ H_20_ O_9_^+^	Hydroxybenzoic acid derivative	Phenolic	275,165, 141, 121	3.16	^109^
91.	7.919	198.0504	199.0577	C_9_ H_10_ O_5_^+^	Syringic acid	Phenolic	141, 97	2.38	^37^
92.	8.464	242.0402	243.0475	C_10_ H_10_ O_7_^+^	3-Galloyl-glycerinaldehyd	Phenolic	218, 169	2.47	^37^
93.	9.698	438.1503	439.1574	C_21_ H_26_ O_10_^+^	Fortuneanoside B	Phenolic	409, 207	2.3	^37^
94.	11.043	240.061	241.0683	C_11_ H_12_ O_6_^+^	Lignicol	Phenolic	227, 187	2.4	^37^
95.	11.221	378.1289	379.1362	C_19_ H_22_ O_8_^+^	Oleuropein aglycone	Phenolic	294, 207, 100	2.52	^37^
96.	2.322	170.0207	169.0134	C_7_ H_6_ O_5_^−^	Gallic Acid	Phenolic	125	0.81	^110^
97.	2.727	524.1143	523.1072	C_23_ H_24_ O_14_^−^	3,5-di-O-(3-O-methyl galloyl) quinic acid	Phenolic	191, 169	2.31	^111^
98.	2.929	512.1145	511.1073	C_22_ H_24_ O_14_^−^	Galloyl-syringic acid-hexoside I	Phenolic	313, 196, 125	2.08	^112^
99.	4.925	302.0987	301.0914	C_13_ H_18_ O_8_^−^	Tachioside	Phenolic	179, 161,139, 121	1.5	^113^
100.	5.72	154.0259	153.0186	C_7_ H_6_ O_4_^−^	Dihydroxybenzoic acid II	Phenolic	109	0.72	^71^
101.	6.696	302.0622	301.0549	C_12_ H_14_ O_9_^−^	Pyrogallol-2-O-glucuronide	Phenolic	175, 168, 140	1.58	^114^
102.	7.422	342.094	341.0867	C_15_ H_18_ O_9_^−^	Caffeic acid-O-glucoside	Phenolic	179, 135	1.1	^115^
103.	7.595	708.1864	707.1791	C_39_ H_32_ O_13_^−^	Caffeoylquinic acid dimer	Phenolic	353, 191	-2.06	^116^
104.	8.111	474.1349	473.1275	C_20_ H_26_ O_13_^−^	Caffeic acid pentosyl hexoside	Phenolic	443, 353, 341, 179	2.44	^117^
105.	8.187	316.1143	315.1069	C_14_ H_20_ O_8_^−^	vanilloloside	Phenolic	281, 137	1.56	^118,119^
106.	8.411	516.1458	515.1379	C_22_ H_28_ O_14_^−^	3-O-(β-Dglucopyranosyl)- caffeoyl quinic acid	Phenolic	353, 285, 173	2.16	^109,120^
107.	8.535	184.0363	183.029	C_8_ H_8_ O_5_^−^	Methyl gallate	Phenolic	167, 124	0.9	^37^
108.	8.632	386.0831	385.0758	C_16_ H_18_ O_11_^−^	O-Feruloylgalactaric acid I	Phenolic	353, 183	1.83	^121^
109.	8.954	526.1296	525.1223	C_23_ H_26_ O_14_^−^	Galloyl-eudesmic acid-hexoside I	Phenolic	353, 341, 183, 168	2.68	^112^
110.	9.007	684.1872	683.18	C_30_ H_36_ O_18_^−^	Rosmarinic acid-di-O-hexoside	Phenolic	521, 359, 341	2.95	^122^
111.	9.109	326.0987	325.0914	C_15_ H_18_ O_8_^−^	4-O-Glucosylcinnamic acid	Phenolic	293, 163	1.45	^123^
112.	9.308	448.1563	447.1491	C_19_ H_28_ O_12_^−^	Apigenin derivative	Phenolic	401, 293, 269, 161, 101	1.76	^124^
113.	9.95	306.0363	305.0291	C_14_ H_10_ O_8_^−^	Methyl brevifolincarboxylate	Phenolic	273,245,221,217,187,000	1.23	^125^
114.	9.987	462.172	461.1647	C_20_ H_30_ O_12_^−^	Rebouoside C	Phenolic	299, 137	1.76	^126^
115.	10.195	374.1561	373.1487	C_17_ H_26_ O_9_^−^	Decenedioyl quinic acid	Phenolic	199, 173, 125	1.59	
116.	10.317	302.0047	300.9974	C_14_ H_6_ O_8_^−^	Ellagic Acid	Phenolic	573, 483	1.57	^37^
117.	10.504	516.1241	515.1169	C_25_ H_24_ O_12_^−^	Dicaffeoyl-quinic acid	Phenolic	353, 335, 191, 179, 173, 161, 135	2.64	^127^
118.	10.724	448.0982	447.091	C_21_ H_20_ O_11_^−^	Quercetin 3-deoxyhexoside	Phenolic	303, 265	2.36	^128^
119.	11.316	560.1509	559.143	C_27_ H_28_ O_13_^−^	1-O-caffeoyl-3-O-sinapoylquinic acid	Phenolic	559, 381, 193	2.13	^129^
120.	12.034	210.0911	209.0842	C_11_ H_14_ O_4_^−^	Sinapyl alcohol	Phenolic	176, 112, 91	-1.92	^130^
121.	12.08	538.166	537.1588	C_25_ H_30_ O_13_^−^	Coumaroyl-dihydromonotropein	Phenolic	465, 238, 112, 91	2.66	^131^
122.	12.271	178.0618	177.0549	C_10_ H_10_ O_3_^−^	4-Methoxycinnamic acid	Phenolic	163, 149	1.19	^37^
123.	13.45	344.0514	343.0442	C_17_ H_12_ O_8_^−^	Tri-O-methylellagic acid	Phenolic	328, 312, 297, 285, 269	1.78	^91^
124.	9.159	292.0205	291.0133	C_13_ H_8_ O_8_^−^	Brevifolincarboxylic acid	Phenolic	243, 194	1.39	^37^
125.	9.241	368.1085	367.1015	C_17_ H_20_ O_9_^−^	3-Feruloylquinic acid	Phenolic	191, 173	2.26	^132^
126.	9.345	180.0414	179.0341	C_9_ H_8_ O_4_^−^	Caffeic acid	Phenolic	135, 134, 117, 107, 89	0.81	^133^
127.	9.699	336.0832	335.076	C_16_ H_16_ O_8_^−^	5-O-Caffeoylshikimic acid	Phenolic	179, 161, 135	1.31	^133^
128.	14.582	294.1817	293.1744	C_17_ H_26_ O_4_^−^	6-Gingerol	Phenolic	277, 221, 177	1.42	^134^
Sterols									
129.	15.432	578.2728	577.2659	C_30_ H_42_ O_11_^−^	Hellebrigenin glucoside	Sterols	415	-0.1	^135^
130.	15.935	392.2908	391.2834	C_24_ H_40_ O_4_^−^	Deoxycholic acid	Sterols	391, 337, 297, 279	1.85	^136^
131.	16.473	432.226	431.2188	C_28_ H_32_ O_4_^−^	Sterostrein A	Sterols	413, 387	4.11	^37^
132.	20.878	480.3795	479.3721	C_29_ H_52_ O_5_^−^	Unknown sterol	Sterols	381, 125, 100	1.99	
133.	21.573	462.3694	461.3623	C_29_ H_50_ O_4_^−^	Unknown sterol	Sterols	409, 144, 100	1.55	
134.	21.927	448.3534	447.3459	C_28_ H_48_ O_4_^−^	Typhasterol	Sterols	384, 125, 100	1.83	
135.	21.141	588.4212	589.4285	C_39_ H_56_ O_4_^+^	Stigmasteryl ferulate	Sterols	575, 487, 394	-3.35	^37^
136.	23.515	396.3755	397.3827	C_29_ H_48_^+^	Stigmasta-3,5-diene	Sterols	335, 144, 68	0.15	^37^
Sugar Derivatives									
137.	0.993	384.1256	383.1184	C_14_ H_24_ O_12_^−^	Acetyl-maltose	Sugar derivatives	357,365, 191	1.17	^106^
138.	1.034	534.1774	533.1702	C_19_ H_34_ O_17_^−^	Glucopyranosyl fructofuranosi de quinic acid	Sugar derivatives	383, 353, 191,	2.16	^136^
139.	2.016	162.0521	161.0448	C_6_ H_10_ O_5_^−^	Meglutol	Sugar derivatives	159, 141, 123	0.74	^37^
140.	8.771	294.1301	293.1228	C_12_ H_22_ O_8_^−^	2-O-glucosyl-hexanoic acid	Sugar derivatives	248, 183, 153	1.36	^113^
141.	9.779	382.1821	381.1748	C_16_ H_30_ O_10_^−^	Methylbutyl-O-pentosylhexoside	Sugar derivatives	337, 305	1.82	^15^
142.	1.331	342.1144	341.1072	C_12_ H_22_ O_11_^−^	Sucrose	Sugars	178, 160	1.77	^137^
Terpenoids									
143.	17.321	434.2413	433.2341	C_28_ H_34_ O_4_^−^	20-β-Hydroxyscutione	Terpenoid	238, 152, 112	4.39	^138^
144.	9.38	390.1511	389.1437	C_17_ H_26_ O_10_^−^	Loganin	Terpenoids	327, 267	1.53	^28^
145.	10.517	426.187	425.1799	C_21_ H_30_ O_9_^−^	Abscisic acid-hexose	Terpenoids	263, 153	1.94	^139^
146.	11.245	514.2394	513.2315	C_25_ H_38_ O_11_^−^	Taxchinin J	Terpenoids	323, 263, 221, 179, 161, 125	1.98	^140^
147.	11.436	538.3329	537.3256	C_33_ H_46_ O_6_^−^	unknown triterpenoid	Terpenoids	463, 389, 238, 91	-3.44	
148.	13.53	406.2694	405.2625	C_24_ H_38_ O_5_^−^	3α,12α-dihydroxy-7-oxo-5β-cholanic acid	Terpenoids	343, 361, 289	2.55	^141^
149.	14.239	408.2856	407.2783	C_24_ H_40_ O_5_^−^	Cholic acid	Terpenoids	311, 238, 112, 107	2	^142^
150.	14.84	488.3479	487.3406	C_30_ H_48_ O_5_^−^	Asiatic Acid	Terpenoids	453, 417	2.24	^37^
151.	15.586	266.1536	265.1468	C_15_ H_22_ O_4_^−^	Deoxyartemisinin	Terpenoids	213, 100	-1.79	^37^
152.	16.679	556.2886	555.2816	C_28_ H_44_ O_11_^−^	Forskoditerpenoside C	Terpenoids	521, 485	-0.22	^143^
153.	19.403	456.3585	455.3512	C_30_ H_48_ O_3_^−^	Betulinic acid	Terpenoids	327, 317, 353,409, 437	1.89	^144^
154.	20.763	472.3534	471.3462	C_30_ H_48_ O_4_^−^	Maslinic acid	Terpenoids	423, 393, 405	1.85	^145^
155.	22.494	608.4261	607.4188	C_35_ H_60_ O_8_^−^	Sporminarin B	Terpenoids	544, 355, 316, 125, 100	2.72	
156.	22.674	620.4261	619.4188	C_36_ H_60_ O_8_^−^	Fasciculic acid A	Terpenoids	581, 545, 527	2.71	^37^
157.	8.918	388.1707	389.1779	C_18_ H_28_ O_9_^+^	Tuberonic acid	Terpenoids	207, 163, 89	2.64	^133^
158.	10.743	378.1652	379.1727	C_20_ H_26_ O_7_^+^	Cnicin	Terpenoids	247, 229, 211, 155, 129	2.6	^146^
159.	14.816	510.3319	511.339	C_32_ H_46_ O_5_^+^	Ganodermic acid TQ	Terpenoids	453, 365, 239, 100	2.65	^37^
160.	14.829	452.3287	453.3361	C_30_ H_44_ O_3_^+^	Ganoderic acid SZ	Terpenoids	351, 262	0.3	^37^

**Fig. 4:**
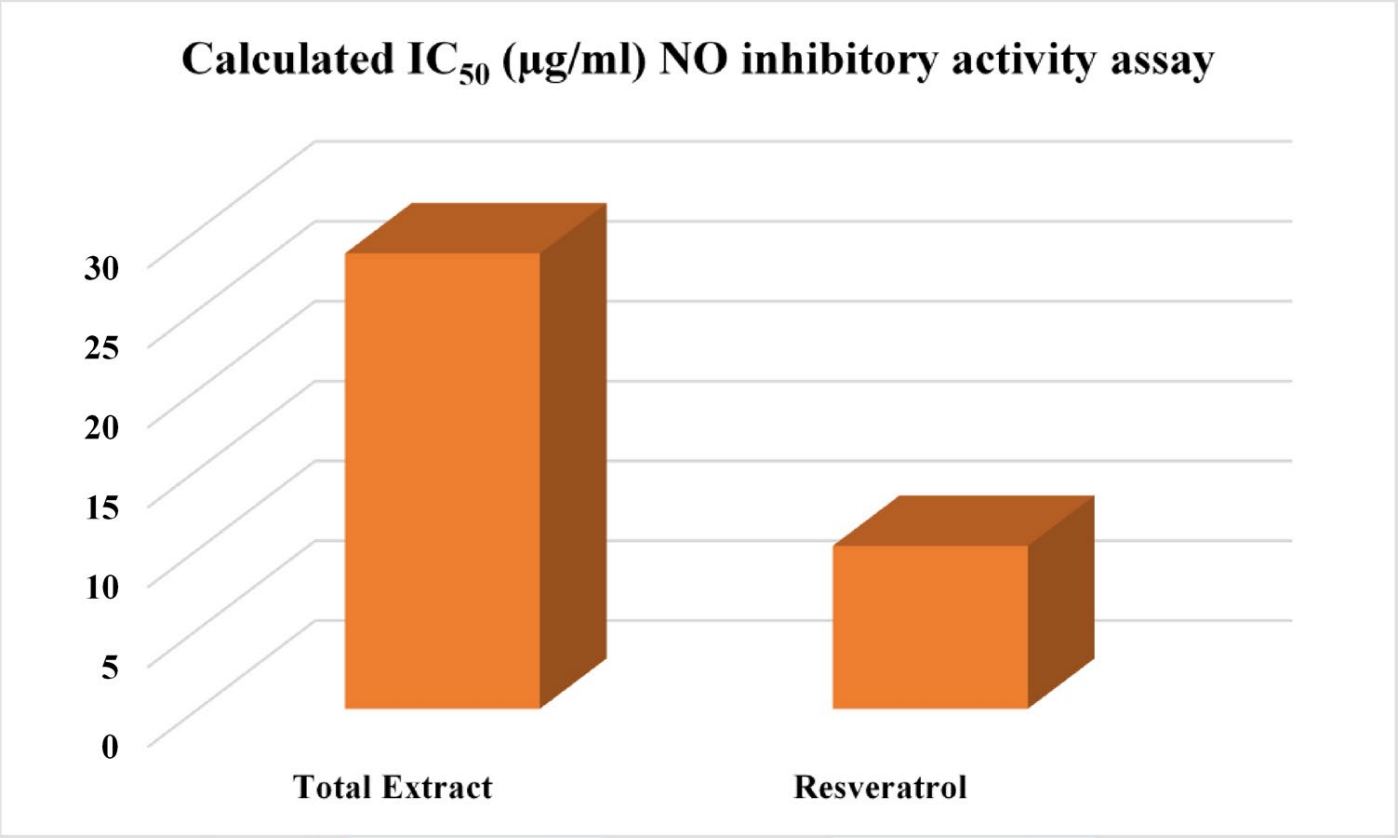
Calculated IC_50_ (µg/ml) of *F. natalensis* fruits methanolic extract of and Resveratrol by NO inhibitory activity assay.

### Phenolic compounds

UPLC-MS/MS profiling of *F. natalensis* fruit revealed 41 phenolic metabolites, belonging to hydroxybenzoic and hydroxycinnamic acids, galloyl derivatives, lignans, and complex caffeoylquinic esters. Several bioactive compounds such as, gallic acid (peak 96; m/z 169.0134 [M-H]^−^ C_7_H_6_O_5_^−^) and ellagic acid (peak 116; m/z 301.0064 [M-H]^−^ C_14_H_6_O_8_^−^) were identified. Caffeic acid (m/z 179.0341 [M-H]^−^ C_9_H_8_O_4_^−^) and syringic acid (m/z 193.050 [M-H]^−^ C_9_H_10_O_5_^−^), were annotated. Such phenolic compounds are known for potent antioxidant and anti-inflammatory properties via scavenging reactive oxygen species and modulating the NF-κB signaling pathway^19^. The presence of gallic and ellagic acids, common in the Moraceae family, in accordance with previous reports on *F. carica* and *F. religiosa*^20^. Moreover, gallic acid, methyl gallate, syringic acid, and ellagic acid were reported in *F. carica* fruits^21^, with several biological properties including antioxidant, antidiabetic, and antimicrobial^22^. Phenolic compounds such as rosmarinic acid-di-*O*-hexoside (peak 110, m/z 683.1801 [M-H]^−^), dicaffeoylquinic acid (peak 117, m/z 515.1169 [M-H]^−^), and 1-*O*-caffeoyl-3-*O*-sinapoylquinic acid (peak 117, m/z 515.1169 [M-H]^−^) were detected in *F. natalensis* fruits. Such results are consistent with the rich content of phenolic compounds in *F. benghalensis* and *F. religiosa*^23^. The caffeoylquinic derivatives are known to enhance antioxidant capacity and modulate inflammatory pathways^24^. The phenolic compounds of *F. natalensis* fruit are structurally varied compared to the previously reported for other *Ficus* fruits, which revealed their potent antioxidant, anti-inflammatory, and therapeutic potential. The detection of oleuropein aglycone, a phenolic compound abundantly found in olive fruit with myriad of biological activities^25^, further valorizes the phytochemical and pharmacological significance of *F. natalensis* fruit. The detection of several hydroxycinnamic and hydroxybenzoic acids in *F. natalensis* aligns with reports of widespread distribution of these metabolites in the Ficus genus^26^. In accordance with previous reports, phenolic compounds were enriched in *F. deltoidei*, *F. drupacea*, and *F. sycomorus*^16^.

### Flavonoids

Flavonoids are a diverse group of natural polyphenolic compounds widely distributed in vegetables, fruits, and grains^27^. As major plant secondary metabolites, they play pivotal roles in human health as they constitute one of the most significant groups of bioactive phytochemicals in human diet and with potential antioxidant, antimicrobial, anti-inflammatory, and cytoprotective effects^27^. A total of 21 flavonoids were annotated in *F. natalensis* fruit with both aglycones and glycosidic derivatives. In the negative ionization mode, quercetin (peak 60; m/z 301.0340 [M-H]^−^) and its glycosides such as quercetin-3-*O*-glucoside (isoquercitrin) (peak 58; m/z 463.089 [M-H]^−^) were identified. Similarly, kaempferol-dihexoside (peak 57; m/z 609.1433 [M-H]^−^) exhibited a characteristic neutral loss of 308 amu, corresponding to the sequential elimination of two hexose moieties, thereby confirming its di-glycosylated structure^28^. Quercetin, kaempferol, and isorhamnetin glycosides such as isoquercetin, avicularin, and quercetin-3-*O*-pentosylhexoside were identified. The detection of these flavonoids agrees with earlier reports on *F. carica*, *F. sycomorus*, and *F. benghalensis*, where these flavonols dominated and were linked to strong antioxidant and anti-inflammatory activities^26^. The diversity of flavonoid glycosides identified here highlights the metabolic richness of *F. natalensis* and underscores its potential as a dietary source of bioactive flavonoids. In accordance with previous studies, LC-MS/MS was applied for profiling of three *Ficus* species including *F. deltoidei*, *F. drupacea* and *F. sycomorus*^16^ revealing the annotation of 90 metabolites belonging to phenolic acids, flavonoids, furanocoumarins, fatty acids and terpenoids^16^. UPLC-MS profiling of *F. benghalensis* aerial root revealed identification of 84 metabolites belonging to isoflavonoids, triterpenes, sterols, fatty acids, cyclic peptides, apocarotenoids, hydroxybenzoates, and hydroxycinnamates^17^. The metabolite profiles of *F. lyrata* leaves and fruits grown in Egypt were performed using UPLC-MS/MS with identification of 72 metabolites belonging to flavonoids, phenolic acids and fatty acids^18^.

### Organic and phenolic acids

A total of 26 organic acids were identified in *F. natalensis* fruit by UPLC-MS/MS, comprising aliphatic, hydroxy, dicarboxylic, and phenolic acids. Citric acid (peak 7; m/z 191.0190 [M-H]^−^), malic (peak 6; m/z 133.0137 [M-H]^−^), succinic (peak 26; m/z 119.0337 [M + H]^+^), and quinic acid (peak 5; m/z 191.0553 [M-H]^−^) were identified. Moreover, abscisic (peak 19) and geniposidic acids (peak 22) were detected. The organic acid profile of *F. natalensis* fruit revealed by UPLC-MS/MS aligns closely with those reported for other Ficus species^16^. The predominance of citric, malic, and quinic acids play a pivotal role in regulation of fruit acidity and flavor as previously reported in *F. carica* and *F. sycomorus*^29^. However, *F. natalensis* showed a richer diversity of dicarboxylic acids, including azelaic and sebacic acids, which are less commonly reported in *Ficus* fruits. Phenolic acids including 3-*O*-caffeoylquinic (peak 3; m/z 353.063 [M-H]^−^)^30^, vanillic (peak 14; m/z 167.0342 [M-H]^−^), p-hydroxybenzoic (peak 15; m/z 137.0235[M-H]^−^), and ferulic acids (peak 18; m/z 193.0561[M-H]^−^), together with their glycosides were identified. Phenolic acids are well known for their biological importance including antioxidant and anti-inflammatory activities^31^. The detection of phenolic acids such as caffeoylquinic, ferulic, and vanillic acids is consistent with earlier findings in *F. benghalensis* and *F. racemosa*^32^.

### Coumarins

Three coumarin derivatives were identified in *F. natalensis* fruit, including maclurin (peak 27; m/z 263.0561 [M + H]^+^), pteryxin (peak 29; m/z 385.127 [M-H]^−^), and 5-*O*-β-D-glucopyranosyl-6-hydroxyangelicin (peak 28; m/z 381.0799 [M + H]^+^) which was characterized by a diagnostic aglycone ion at m/z 219 after sugar loss. Coumarins are known for their antithrombotic, hepatoprotective, and anti-inflammatory activities mediated through suppression of prostaglandin synthesis^33^. These compounds are frequently reported in *Ficus* spp. and have been associated with potential health effects^34^. Their occurrence supports the ethnopharmacological relevance of *F. natalensis*, particularly in traditional remedies for infections and vascular disorders. The detection of *p*-coumaroylquinic acid (m/z 337.0915 [M-H]^−^) further emphasizes the coumarin-phenolic interface typical of *Ficus* species^35^. In accordance with previous studies, furanocoumarins were identified in *F. deltoidei*, *F. drupacea* and *F. sycomorus*^16^.

### Fatty acids and esters

Several fatty acids were identified in *F. natalensis* using UPLC-MS/MS including linoleic, palmitic, and oleic acids. A diverse profile of 22 fatty acids and related esters was detected in *F. natalensis* fruit, encompassing hydroxylated, polyunsaturated, and conjugated species such as linolenic acid, stearidonic acid, ricinoleic acid, colnelenic acid, and dihydrojasmonic acid. Hydroxylated fatty acids including 13-hydroxyoctadeca-9,15-dienoic acid and dihydroxyhexadecanoic acid are known oxylipin intermediates were also identified. Unsaturated fatty acids such as linolenic acid (m/z 279.2319 [M + H]^+^) and stearidonic Acid (m/z 277.2161 [M + H]^+^) were detected. Linolenic and stearidonic acids are ω−3 polyunsaturated fatty acids (PUFAs) that act as precursors for oxylipins, a group of lipid mediators with potent anti-inflammatory and cardioprotective effects^36^. Colneleic (peak 40; m/z 295.2262 [M + H]^+^) and colnelenic acids (peak 38; m/z 293.2107 [M + H]^+^)^37^, are oxygenated linoleic acid derivatives were detected in *F. natalensis* fruit. Similarly, the detection of 9,12,13-trihydroxy-10,15-octadecadienoic acid and 16-hydroxy-9-oxo-10,12,14-octadecatrienoic acid indicates lipid oxidation processes yielding trihydroxy and oxo-fatty acids, compounds frequently associated with oxidative stress responses and anti-inflammatory activities. Similar lipid constituents have been reported in other Ficus fruits including *F. carica* and *F. sycomorus*, supporting their nutritional value and cardioprotective potential^38,39^. Fatty acid esters such as ascorbyl palmitate (peak 36; m/z 415.2663 [M + H]^+^) and glyceryl linolenate (peak 50; m/z 353.2692 M + H]^+^) were identified further expands the lipid profile of *F. natalensis*. Ascorbyl palmitate, an esterified form of vitamin C and palmitic acid, acts as a potent lipid-soluble antioxidant^40^. Glyceryl linolenate is a monoacylglycerol derivative previously reported in *F. exasperata* with potential anti-inflammatory activity^41^.

### Iridoids and glycosides

Iridoids, a class of bioactive metabolites widely distributed in plant species with myriad of biological properties including neuroprotective, hepatoprotective, anti-inflammatory, antitumor, hypoglycemic, and hypolipidemic activities^42^. Three iridoid glycosides were identified including 8-*O*-acetylshanzhiside (peak 85; m/z 435.1477 [M + H]^+^; C_18_H_26_O_12_^+^)^43^ and shanzhiside methyl ester (peak 87; m/z 451.1433 [M-H]^−^)^44^. Their fragmentation patterns, including neutral losses of glucose and acetyl moieties, confirmed their structures. Monotropein derivatives (peak 86; m/z 695.2019 [M-H]^−^) showed sequential sugar losses (−162, −180), typical of di-glucosylated iridoids^45^. These metabolites are known for anti-inflammatory and hepatoprotective properties^46^.

A total of 11 glycosides were identified, including coumarin, phenolic, monoterpenoid, and lignan-derived sugar conjugates. Glycosides were primarily represented by iridoid, phenolic, and terpenoid-linked sugars. Umbelliferone hexoside (peak 74; m/z 325.0894 [M + H]^+^) exhibited characteristic fragmentation at m/z 163, 119 corresponding to umbelliferone aglycone^47^. Syringin (peak 76; m/z 373.1494 [M + H]^+^)^48^ and eugenol rutinoside (peak 80; m/z 471.1851 [M-H]^−^)^49^, were identified based on the loss of sugar moieties (162 amu for hexose and 308 amu for rutinoside). Syringin is a phenylpropanoid glycoside, is widely distributed in various plant species with potential health effects including immunomodulatory, anti-tumor, antihyperglycemic, and antihyperlipidemic effects^50^.

### Terpenoids and sterols

A diverse array of terpenoids including 18 compounds were detected in *F. natalensis* fruit, including abscisic acid-hexose, betulinic acid, maslinic acid, asiatic acid, and ganoderic derivatives. Betulinic acid (peak 153; m/z 455.3512 [M-H]^-^) and maslinic acid (peak 154; m/z 471.3462 [M-H]^-^) were detected confirmed the presence of lupane- and oleanane-type triterpenes. Asiatic acid (peak 150; m/z 487.3406 [M-H]^-^) and cholic acid (peak 149; m/z 407.2783 [M-H]^-^) were also observed. The detection of forskoditerpenoside C (peak 152; m/z 555.2816 [M-H]^-^) and taxchinin J (peak 146; m/z 513.2315 [M-H]^-^) suggests the coexistence of diterpenoid glycosides. Pentacyclic triterpenoids such as betulinic, maslinic, and asiatic acids are widespread in Moraceae and are well known for their antioxidant, anti-inflammatory, and cytoprotective activities^51^. Eight sterols were identified in *F. natalensis* fruit, including hellebrigenin glucoside, deoxycholic acid, sterostrein A, typhasterol, stigmasteryl ferulate, and stigmasta-3,5-diene. These compounds reflect the plant’s diverse sterol biosynthetic machinery, contributing to membrane stability and signaling. The occurrence of phytosterols such as stigmasteryl ferulate and stigmasta-3,5-diene agrees with reports in *F. carica*, *F. sycomorus*, and *F. benghalensis* fruits^52^. Stigmasteryl ferulate, a conjugated steryl ester, is known for potent antioxidant and anti-inflammatory activities, while typhasterol, a brassinosteroid intermediate, plays crucial roles in plant growth and stress tolerance. The sterol profile of *F. natalensis* fruit underscores its potential bioactivity as cardioprotective, hypocholesterolemic, and anti-inflammatory health effects, consistent with the reported bioactivity of sterol-enriched Ficus extracts^53^.

### Sugar derivatives

Six sugar-related metabolites were annotated in *F. natalensis* fruit, including common disaccharides (sucrose and acetyl-maltose) and conjugated sugar acids such as glucopyranosyl fructofuranoside quinic acid, 2-*O*-glucosyl-hexanoic acid, and methylbutyl-O-pentosylhexoside. Sucrose was previously reported in *F. carica* and *F. sycomorus* fruits, where it contributes to sweetness and osmo-protection during ripening^54^.

### GNPS molecular networking

GNPS molecular networking organized the detected metabolites into structurally related clusters, enabling annotation propagation and revealing both known compounds and structurally related unknown analogues. The presence of hub nodes and cluster-specific fragmentation patterns supports the reliability of putative annotations and highlights the chemical diversity of *F. natalensis* fruit metabolites (Figure S7- S9). Cluster-based annotation of the GNPS molecular network suggested that the metabolome of *F. natalensis* fruit is dominated by flavonoid glycosides and their acylated derivatives, alongside triterpenoid saponins and diverse phenolic constituents.

### Anti-inflammatory activity

Inflammation is a complex biological defence mechanism initiated by the immune system in response to harmful stimuli such as microbial infections, toxins, physical injury, and chemical irritants^55^. However, when inflammation becomes chronic or excessive, it contributes to the pathogenesis of several degenerative and metabolic disorders, including cancer, Alzheimer’s disease, atherosclerosis, and cardiovascular diseases^37^. Nitric oxide (NO) plays a pivotal role in the inflammatory process, as secreted by endothelial cells, macrophages, and neurons to serve as a vital chemical mediator in immune responses^56^. Under pathological conditions, overproduction of NO contributes to oxidative stress and tissue injury. Therefore, inhibition of excessive NO production has become a target mechanism for evaluating the anti-inflammatory potential of therapeutic agents^57^. Recently, natural products derived from medicinal plants have drawn significant attention as potential sources of anti-inflammatory agents. Various phytochemicals such as phenolics, flavonoids, terpenoids, steroids, and saponins have been reported to exert potent anti-inflammatory activities through multiple mechanisms including inhibition of NO production^58^.

A nitric oxide inhibitory assay was utilized to investigate the anti-inflammatory properties of the methanolic extract from *F. natalensis* fruit. The extract revealed significant anti-inflammatory activity with an IC_50_ value of 28.54 ± 1.66 µg/mL, compared to resveratrol as a reference anti-inflammatory with IC_50_ of 10.21 ± 0.68 µg/mL (Fig. 4). The diverse phytochemical profile of *F. natalensis* notably phenolic acids, flavonoids, terpenoids, and coumarins collectively contribute to the extract’s anti-inflammatory potential. Potent antioxidant, anti-inflammatory, and antibacterial qualities are well-known for several identified metabolites, especially gallic acid and its derivatives^59^. For instance, gallic acid reduces inflammation by preventing the release of cytokines that promote inflammation^60^. Furthermore, substances like betulinic acid, ellagic acid, and quercetin greatly enhance the extract’s bioactivity^37,61^. These findings are consistent with previous studies on other Ficus species, such as *F. benghalensis*, *F. carica*, and *F. religiosa*, which also exhibited significant anti-inflammatory effects^62–64^. Furthermore, *F. natalensis* leaves exhibited a potent anti-inflammatory potential when tested in vitro and in vivo^65^. These results highlight the therapeutic potential of *F. natalensis* fruit extract as a natural source of anti-inflammatory agents. Recent studies have proved several pharmacological effects of Ficus species^7^. For instance, the ethanol extract of *F. racemosa* leaf showed anti-inflammatory activity by inhibiting the formation of prostaglandins PGE2 and PGD2, 5-lipoxygenase (IC_50_ = 58 µg/ml), and decreased the activity of COX-1 (IC_50_ = 83 µg/ml)^7^. *F. natalensis* leaf extract can attenuate CdCl_2_-induced testicular impairments by inhibiting oxidative cell damage and inflammation^11^.

## Conclusion

The present study provides a comprehensive phytochemical profiling of *Ficus natalensis* fruit using UPLC-MS/MS, alongside an evaluation of its anti-inflammatory potential. A total of 160 metabolites were tentatively annotated, representing diverse phytochemical classes, with phenolics and flavonoids being the predominant constituents. The fruit extract demonstrated notable anti-inflammatory activity (IC_50_ = 28.54 µg/mL) compared to resveratrol (IC_50_ = 10.21 µg/mL), supporting its potential as a source of bioactive compounds. These findings highlight the chemical richness of *F. natalensis* fruit and underscore its potential value as a natural source of pharmacologically relevant metabolites. However, metabolite identification in this study was based on accurate mass measurements and MS/MS fragmentation data and thus remains putative (MSI Level 2) in the absence of validation using authentic reference standards. In addition, isomeric compounds cannot be excluded due to similarities in fragmentation patterns. While UPLC-HRMS is highly effective for qualitative metabolite profiling, definitive structural elucidation and absolute quantification require complementary techniques such as co-analysis with authentic standards and nuclear magnetic resonance (NMR) spectroscopy. Furthermore, although molecular networking and spectral similarity approaches supported metabolite annotation, more advanced metabolomics workflows, including feature-based molecular networking and extensive spectral library matching, would further enhance annotation confidence. From a biological perspective, the current study focused on anti-inflammatory activity; therefore, additional investigations encompassing antioxidant, antimicrobial, enzyme inhibitory, and cytotoxic assays, as well as in vivo studies, are warranted to fully elucidate the pharmacological potential and underlying mechanisms of action of *F. natalensis* fruit. Overall, this work provides a valuable foundation for future phytochemical and pharmacological studies, while emphasizing the need for further validation and comprehensive biological evaluation to substantiate the therapeutic potential of this species.

## Materials and methods

### Plant material

The fruits of *F. natalensis* Hochst. were collected from the Horticultural Research Institute in Giza, Egypt in October 2024. A voucher specimen with the number “ERU-PH-01-Fn/2024”, has been deposited at the Pharmacy Department of the Faculty of Pharmacy, Egyptian Russian University. The plant was botanically verified by Dr. Rim Hamdy, Department of Plant Taxonomy, Faculty of Science, Cairo University.

### Chemicals

MilliQ water supplied by a Millipore MR3 purifier system was used for UPLC analysis. Acetonitrile (J. T. Baker, Deventer, The Netherlands, LC-MS grade, purity ≥ 99.5%) and formic acid (J. T. Baker, Deventer, The Netherlands, LC-MS grade, purity ≥ 99%). All chemicals and standards were purchased from Sigma-Aldrich (St. Louis, MO, USA).

### Samples extraction and preparation for UPLC-HRMS/MS analysis

Freeze-dried *F. natalensis* fruits (*n* = 3 each) as biological replicates were lyophilized and ground in liquid nitrogen using a pestle and mortar. The extraction procedure was conducted as previously described in^25^. About 100 mg of the powdered samples was mixed with 6 mL 100% methanol containing 10 µg/mL umbelliferone as an internal standard (Sigma-Aldrich, St. Louis, MO, USA, purity ≥ 98.0%) and homogenized with an Ultra-Turrax (IKA, Staufen, Germany) at 11,000 rpm, 5 × 60 s with 1 min break intervals. Extracts were then vortexed for 1 min, centrifuged at 3000 g for 10 min, and filtered through a 22 μm pore size filter.

### UPLC-HRMS/MS nalysis

Chromatographic separation of the metabolites was performed using a previously validated method^25^. An Acquity Waters ultra-performance liquid chromatography (UPLC) system (Waters Corp., Milford, MA, USA) equipped with an HSS T3 column (100 × 1.0 mm, particle size 1.8 μm; Waters Corp.) was employed. A gradient elution system was followed at a flow rate of 150 µL/min, starting from formic acid in water (0.1%): CH_3_CN = 95:5–100% CH_3_CN within 35 min, then isocratically for a further 10 min; flow rate 70 µL/min. The electrospray ionization (ESI) source was operated under the following parameters: electrospray voltage, 4.0 kV; capillary temperature, 275 °C; sheath gas, N_2_.

### Metabolite annotation

HRMS data were processed using MS Hunter software for peak alignment, molecular feature extraction, molecular formula prediction, and MS/MS fragment analysis, owing to its robust handling of high-resolution mass data and reliable qualitative annotation capabilities. Metabolites were putatively characterized based on accurate mass measurements, retention behaviour, isotopic pattern matching, and MS/MS fragmentation patterns, through comparison with the reference literature. Metabolite annotation levels were assigned in accordance with the guidelines of the Metabolomics Standards Initiative (MSI). Isotopic pattern similarity was incorporated during formula prediction using MS Hunter, and annotations were accepted only when the isotopic pattern fit exceeded 85% and the mass error was below 10 ppm. Signals exhibiting mass deviations greater than 10 ppm were manually re-evaluated and excluded when confidence was insufficient.

### GNPS feature-based molecular MS/MS network

Using the Feature-based Molecular Networking (FBMN) workflow (version release_28.2)^18^ on GNPS, a molecular network was created. The resulting aligned list of features were exported in a mgf file besides their feature quantification table in csv format. The values of feature quantification table were uploaded onto the FBMN page of GNPS. MS^2^ spectra were filtered, all MS/MS fragment ions within ± 17 Da of the precursor *m/z*were removed, and only the top 6 fragment ions in the ± 50 Da window through the spectrum were utilized. The precursor and fragment ion masses were both set to 0.02 Da. Edges of the molecular network were filtered to have a cosine score above 0.7 and more than 5 matched peaks between the connected nodes. The edges between two nodes were kept in the network if and only if each of the nodes appeared in each other’s respective top 10 most similar nodes. The size of clusters in the network was set to a maximum of 100.The molecular networks were visualized using Cytoscape 3.9.1^17^. Metabolites were annotated based on molecular formula and their fragmentation pattern, compared to earlier reported data aided with public literatures, libraries, and databases.

### Extraction for biological investigation

About 200 g of dried powdered *F. natalensis* fruits were macerated in methanol at room temperature with stirring. The process was carried out three times until exhaustion. The compiled methanol extract was concentrated under vacuum using Rotary evaporator to yield about 15 g of dried methanol extract.

### Anti-inflammatory activity

NO inhibition activity of the tested samples was determined by using a sodium nitroprusside (SNP) as previously reported^66^. The Greiss reagent was used to detect the nitrite ions that are produced when the NO radical produced by SNP in an aqueous solution at physiological pH combines with oxygen. For 150 min, the reaction mixture (2 mL) in phosphate-buffered saline (PBS; pH 7.4) with different concentrations of the tested samples and SNP (10 mM) was incubated at 25 °C. Following the incubation period, 1 mL of the reaction mixture samples was diluted with 1 mL of Greiss reagent, which consists of 0.1% naphthyl ethylene diamine dihydrochloride and 1% sulphanilamide (w/v) in 5% phosphoric acid (v/v). The mixture was incubated at 25 °C and for 30 min. The absorbance of these solutions was measured at 546 nm against the blank solution (without SNP). Resveratrol was used as a reference standard. All the tests were performed in triplicate. The percent inhibition activity was calculated using the formula.

1 $$\:\mathrm{I}\mathrm{n}\mathrm{h}\mathrm{i}\mathrm{b}\mathrm{i}\mathrm{t}\mathrm{i}\mathrm{o}\mathrm{n}\:\mathrm{\%}\:=\left[\frac{\mathrm{A}\:\mathrm{c}\mathrm{o}\mathrm{n}\mathrm{t}\mathrm{r}\mathrm{o}\mathrm{l}-\mathrm{A}\:\mathrm{s}\mathrm{a}\mathrm{m}\mathrm{p}\mathrm{l}\mathrm{e}}{\mathrm{A}\:\mathrm{c}\mathrm{o}\mathrm{n}\mathrm{t}\mathrm{r}\mathrm{o}\mathrm{l}}\right]\mathrm{x}100$$

where, A_control_ is the absorbance of the control reaction at 546 nm and A_test_ represents the absorbance of a test reaction at the same wavelength. Tested material concentration providing 50% inhibition (IC_50_) was calculated from the graph plotting inhibition percentage against concentration.

### Statistical analysis

The results of biological investigation were analysed in triplicate and displayed as average ± standard deviation of the mean (SD).

## Supplementary Information

Below is the link to the electronic supplementary material.

Supplementary material 1 (DOCX 1233.1 kb)

## Data Availability

All data generated or analysed during this study are included in this published article.
