# Supplementary material for: Profiling metabolites of Ficus natalensis hochst. fruit by UPLC-MS/MS and evaluation of anti-inflammatory activity
Source: Sci Rep. 2026 May 13;16:15041. doi: 10.1038/s41598-026-51688-4 (PMC13172326; doi:10.1038/s41598-026-51688-4)
Supplement: Supplementary file 1 — Supplementary material 1 (DOCX 1233.1 kb) [file 41598_2026_51688_MOESM1_ESM.docx]

**Supplementary Materials**

**Profiling Metabolites of *Ficus natalensis* Hochst. Fruit by UPLC-MS/MS and evaluation of anti-inflammatory activity**

**Enas M. Shawky ^1^, Rim Hamdy^2,3^, Mostafa H. Baky ^1*^**

*^1^ Department of Pharmacognosy, Faculty of Pharmacy, Egyptian Russian University, Badr City, 11829, Cairo, Egypt*

*^2^* *Botany and Microbiology Department, Faculty of Science, Cairo University, Giza, 12613, Egypt*

*^3^ Department of Biological Sciences, Faculty of Science, Galala University, New Galala City, 43511, Suez, Egypt*

*Corresponding authors:

**Mostafa H. Baky**

*Pharmacognosy Department, College of Pharmacy, Egyptian Russian University, Badr City 11829, Cairo, Egypt.*
E-mail addresses: [dr_mostafa1984@yahoo.com](mailto:dr_mostafa1984@yahoo.com) , [mostafa-hasan@eru.edu.eg](mailto:mostafa-hasan@eru.edu.eg)


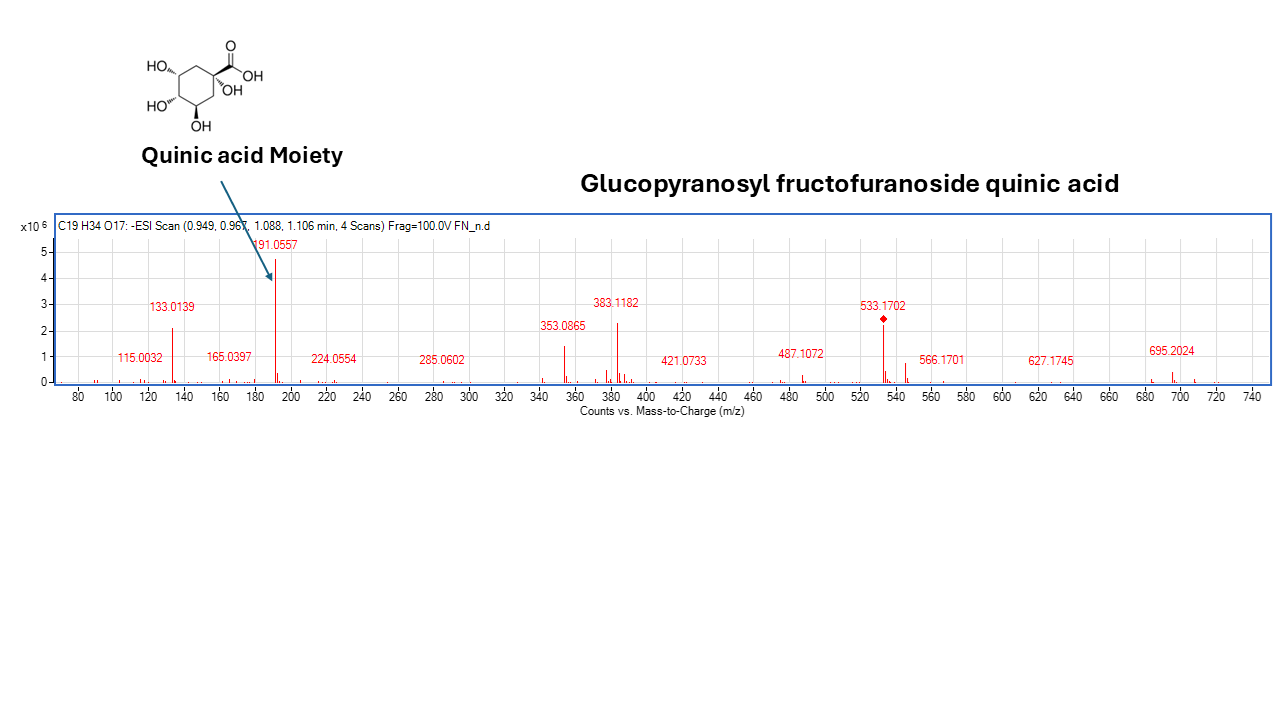


**Figure S1**


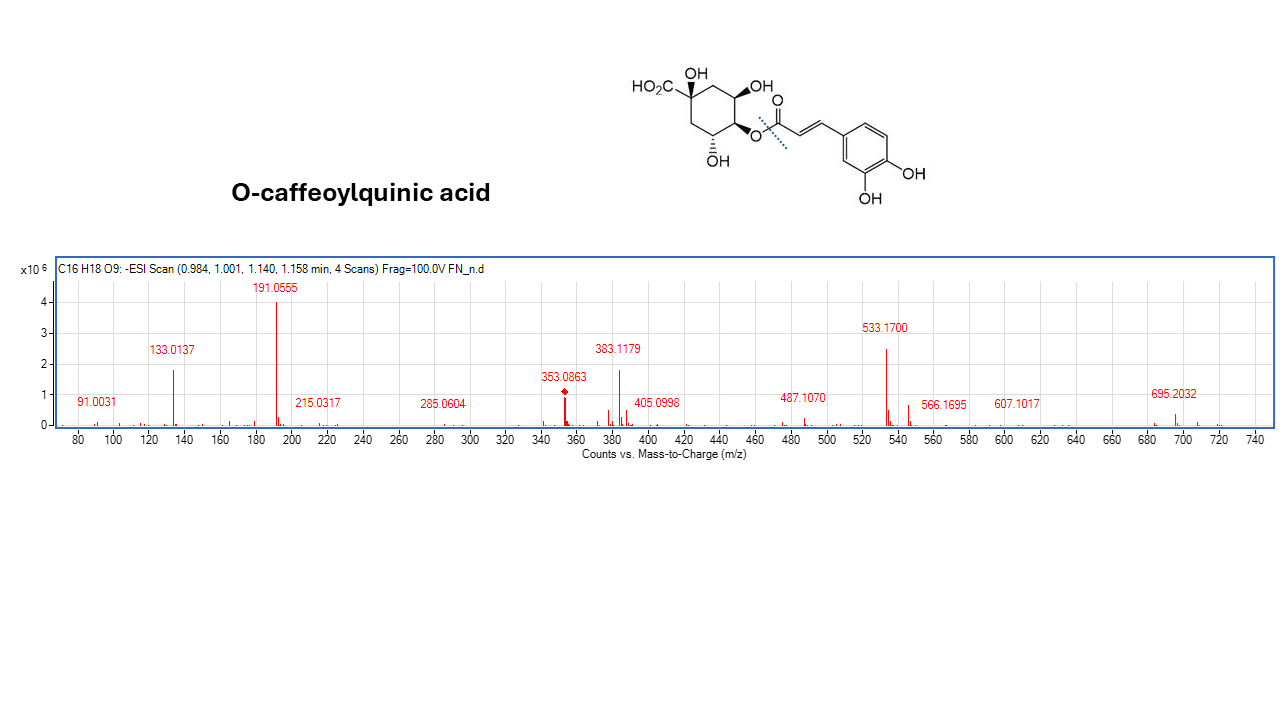


**Figure S2**


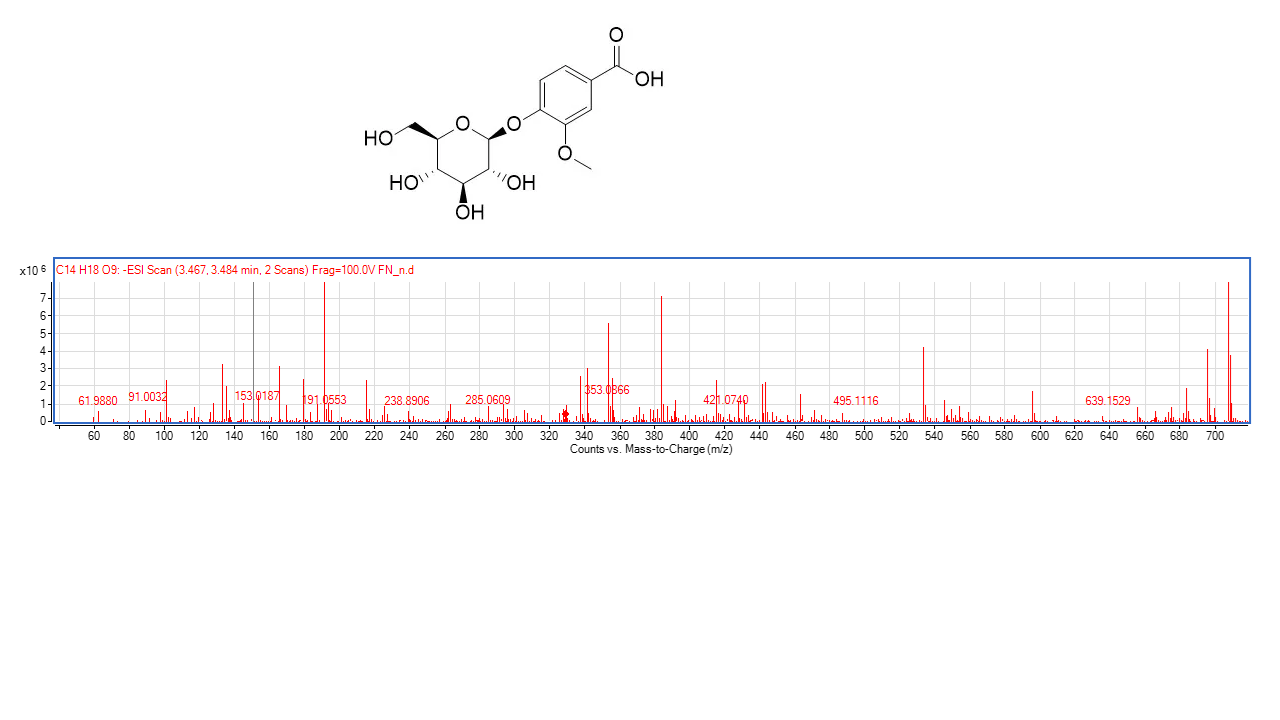


**Figure S3**


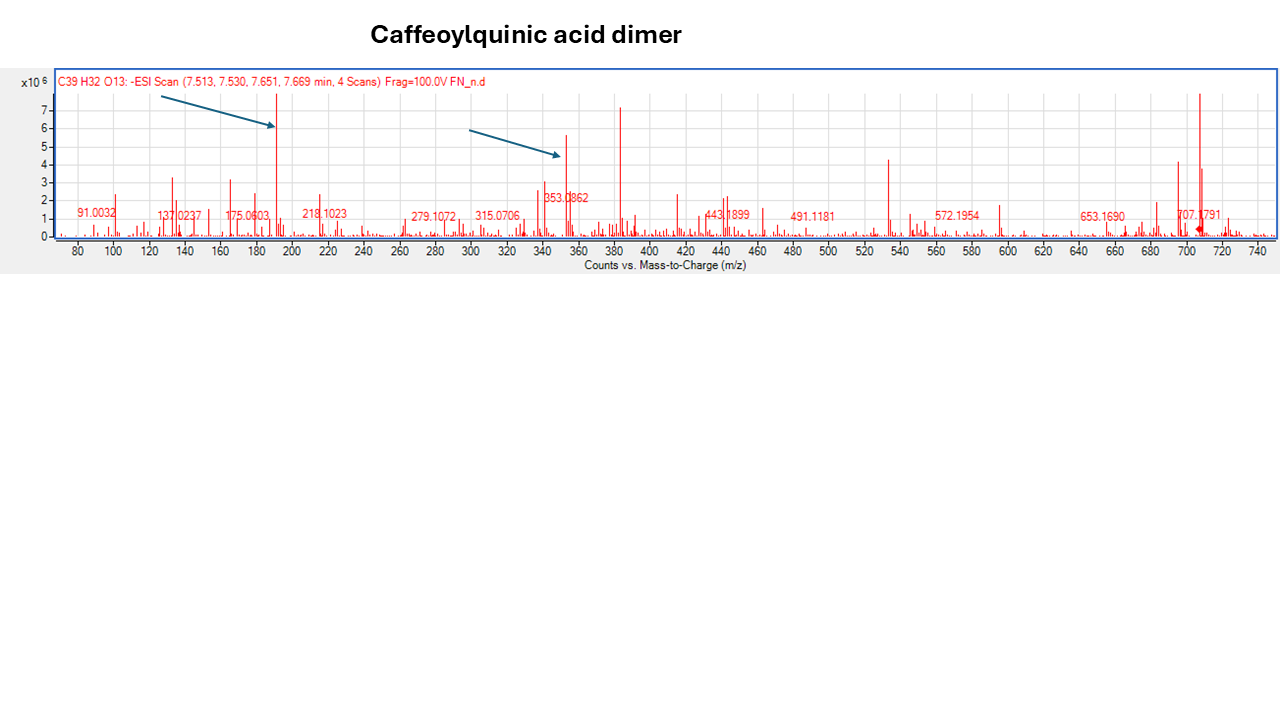


**Figure S4**


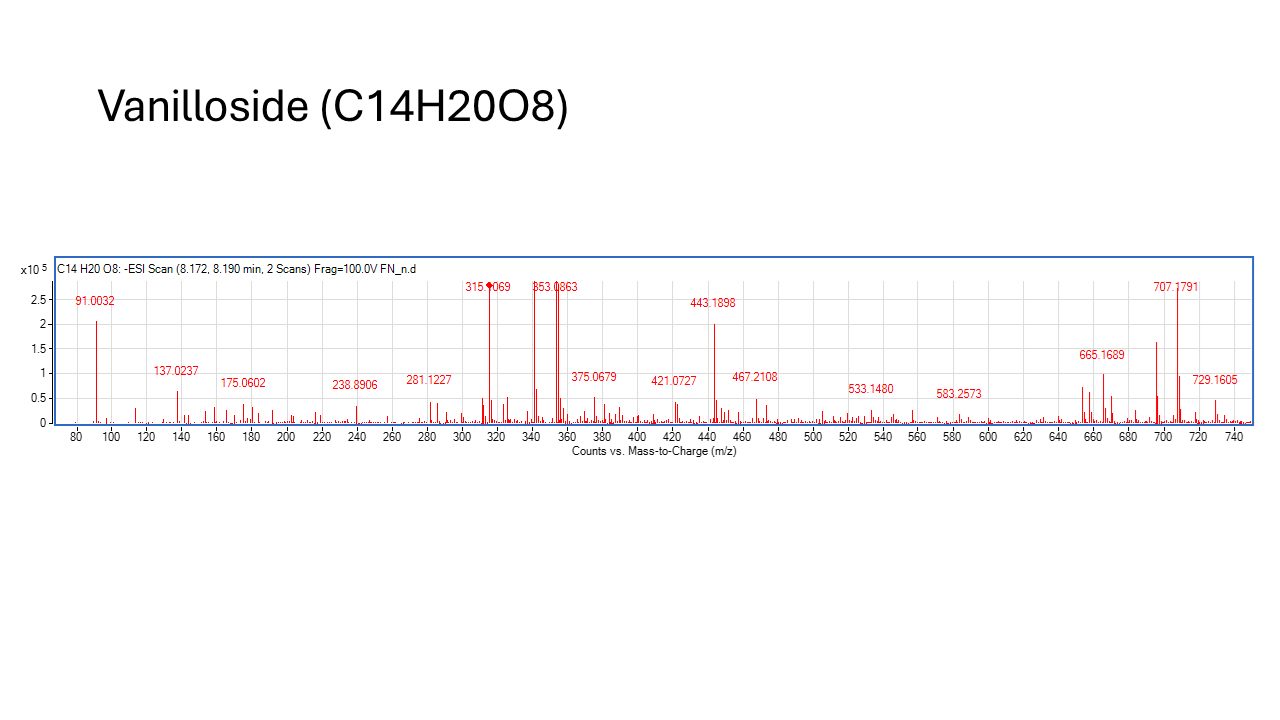


**Figure S5**


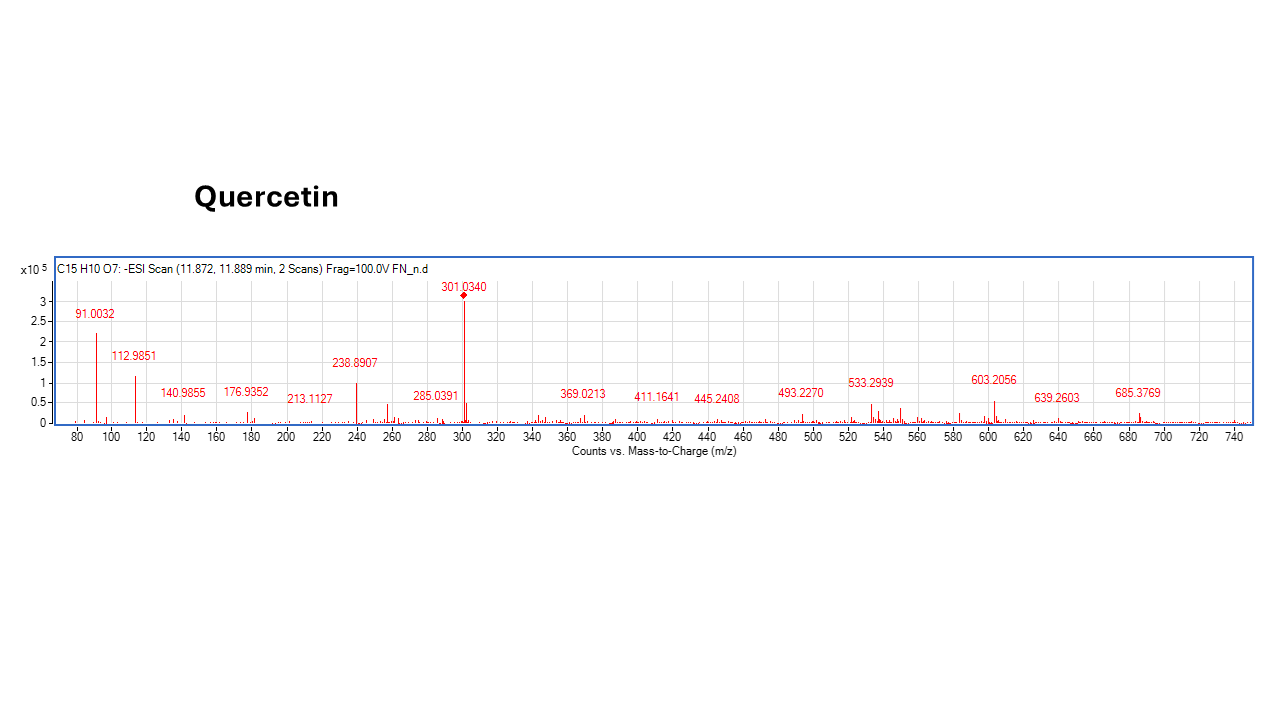


**Figure S6**


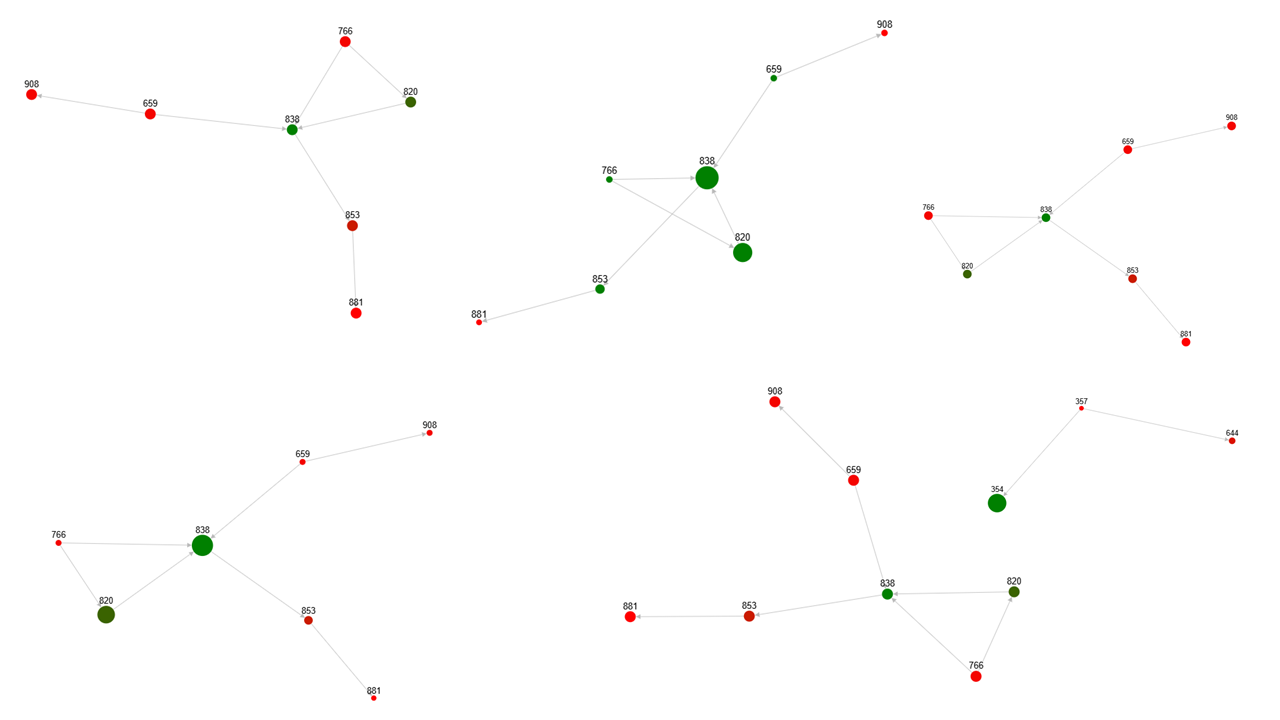


**Figure S7.** GNPS molecular network of metabolites detected in *Ficus natalensis* fruit extract using UPLC–MS/MS.


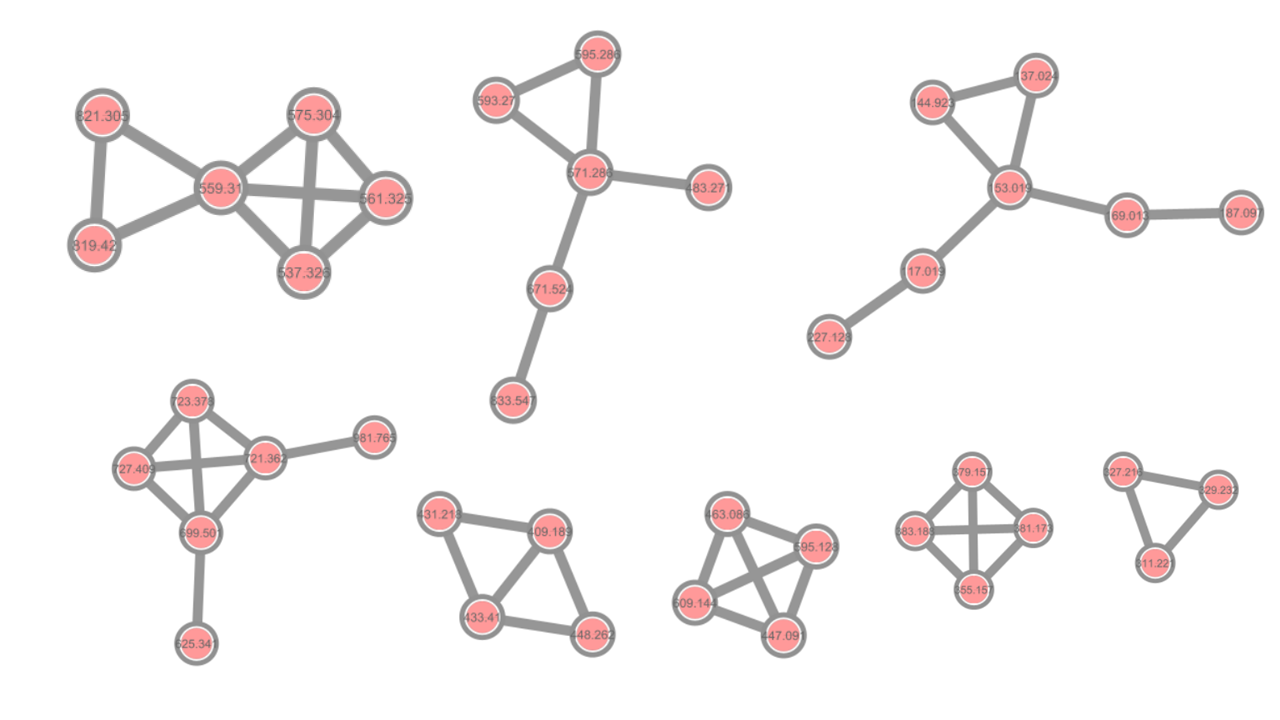


**Figure S8.** GNPS molecular network of Ficus natalensis fruit metabolites labelled by retention time (RT).


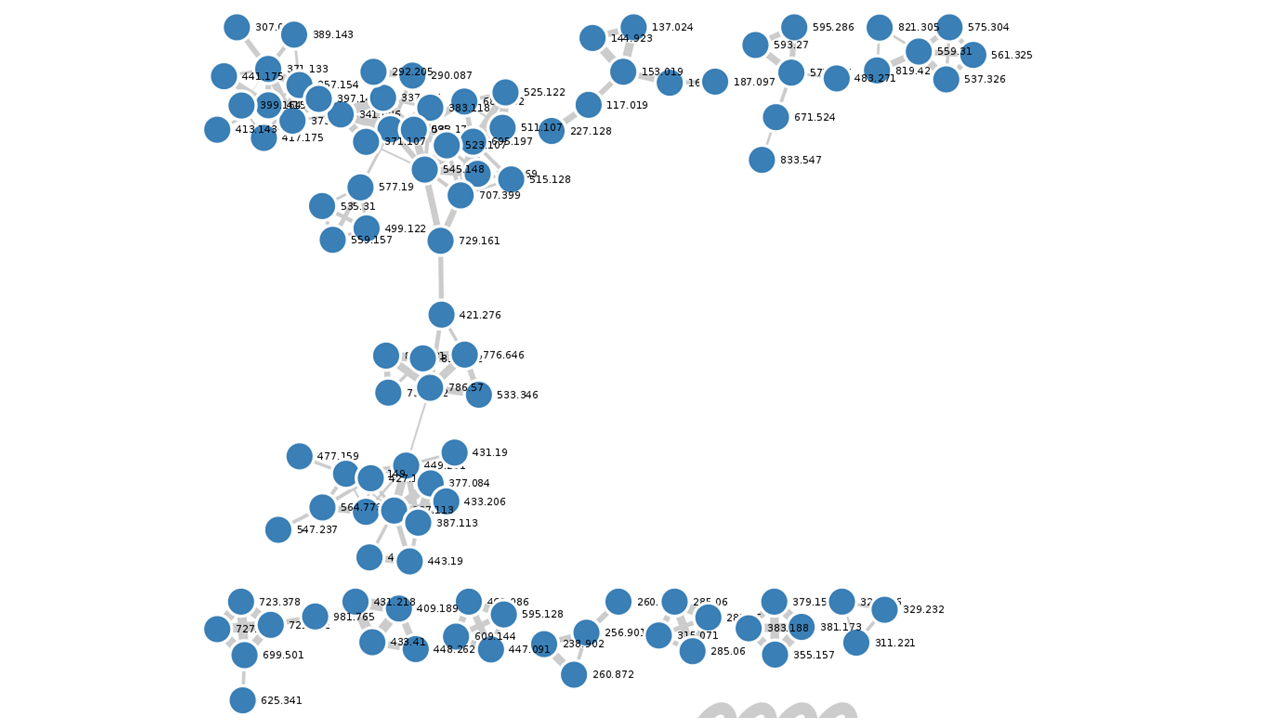


**Fig S9.** GNPS molecular network of Ficus natalensis fruit metabolites
